# Switching from Visibility to Invisibility via Fano Resonances: Theory and Experiment

**DOI:** 10.1038/srep08774

**Published:** 2015-03-05

**Authors:** Mikhail V. Rybin, Dmitry S. Filonov, Pavel A. Belov, Yuri S. Kivshar, Mikhail F. Limonov

**Affiliations:** 1 Ioffe Physical-Technical Institute, St. Petersburg 194021, Russia; 2 University ITMO, St. Petersburg 197101, Russia; 3 Nonlinear Physics Center, Research School of Physics and Engineering, Australian National University, Canberra ACT 0200, Australia

## Abstract

Subwavelength structures demonstrate many unusual optical properties which can be employed for engineering of a new generation of functional metadevices, as well as controlled scattering of light and invisibility cloaking. Here we demonstrate that the suppression of light scattering for any direction of observation can be achieved for a uniform dielectric object with high refractive index, in a sharp contrast to the cloaking with multilayered plasmonic structures suggested previously. Our finding is based on the novel physics of cascades of Fano resonances observed in the Mie scattering from a homogeneous dielectric rod. We observe this effect experimentally at microwaves by employing high temperature-dependent dielectric permittivity of a glass cylinder with heated water. Our results open a new avenue in analyzing the optical response of high-index dielectric nanoparticles and the physics of cloaking.

In the past decade, the study of cloaking and invisibility has attracted a lot of attention^1^. Several approaches for achieving the cloaking regime have been proposed on the basis of metamaterials^1^,^2^,^3^,^4^,^5^,^6^,^7^,^8^,^9^,^10^,^11^,^12^,^13^,^14^, and they employ transformation optics^2^,^3^,^4^,^5^, cancellation of light scattering^6^,^7^,^8^, nonlinear response of multi-shell structures^9^, as well as the use of multilayered plasmonic particles^10^, graphene^11^, and magneto-optical effect with an external magnetic field^12^.

The fact that metamaterials can distort straight light rays is based on the transformation optics and Fermat's principle^2^,^3^. Fermat's principle states that light rays follow not geometrical shortest paths but extremal optical paths these are the geometrical length multiplied by the refractive index of the material. If the refractive index varies in space for non-uniform media the optimal paths are no longer straight lines, but are curved, which may cause invisibility of an object. A simplest cylindrical invisibility device can be achieved by a linear transformation that transforms a cylindrical volume of radius *R*_2_ to cylindrical ring of thickness *R*_2_ − *R*_1_ by equation *r* = *R*_1_ + *r*′(*R*_2_ − *R*_1_)/*R*_2_ within 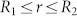 or, equivalently, 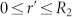. Such transformation medium acts as an invisibility cloak, guiding light around the interior of the cloak and anything placed inside the inner radius *R*_1_ is hidden^15^.

For hiding small subwavelength particles, Alù and Engheta suggested to use plasmonic coatings in order to reduce drastically the total scattering cross-section of an object^16^. The effect is based on the resonant cancellation of the dipole moment of the particle if the polarization vector in a plasmonic shell is antiparallel with respect to that in a dielectric for a given wavelength. To increase the bandwidth of the particle invisibility, it was suggested to cover the particle with several shells of various materials^17^.

The nonlinear response of multi-shell structures was used to study the scattering properties of plasmonic nanoparticles with a nonlinear layer^9^. It was demonstrated that the cloak can be made nonlinear and the cloaking performance of multi-shell plasmonic structures can be controlled by changing the amplitude of the incident wave.

All those approaches require employing specially designed ‘covering shells' with engineered parameters making difficult a practical realization of many invisibility concepts^8^. Here we suggest a novel approach for the realization of tunable invisibility cloaking at all angles of observation that allows a direct switching from visibility to invisibility regimes and back. Our approach is based on the cancelation of scattering from a homogeneous high-index dielectric object *without additional coating layers*. The main idea of our approach is based of the properties of the characteristic lineshape of the Fano resonance^18^, and the cloaking effect is based on the novel physics of the resonant Mie scattering from a homogeneous dielectric rod that appears as cascades of Fano resonances with each individual resonance described by the conventional Fano formula^19^. We demonstrate this novel cloaking effect and its tunability experimentally through a heating-induced change of dielectric permittivity of a glass tube filled with water.

## Results

### General concept and formalism

The well-known Mie scattering is described by analytical solutions of the Maxwell equations for elastic scattering of electromagnetic waves by a sphere^20^,^21^,^22^. If the sphere diameter is comparable with the wavelength of incident light 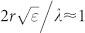, the Mie scattering will be driven by resonances in the dielectric sphere. This leads to the emission of electromagnetic waves by the particle and interference between the nonresonant scattering from the particle and scattering by narrow Mie modes. If a spectrally narrow Mie band interacts constructively or destructively with a broad radiation spectrum, we can expect a Fano-type resonance^18^, a resonant wave phenomenon well-known across many different branches of physics^23^. Some characteristic examples of Fano resonance are found in the studies of magnetization^24^ and electronic polarization properties^25^, semiconductor optics^26^,^27^, electron-phonon coupling in superconductors^28^,^29^,^30^, and scattering in photonic crystals^31^,^32^ and plasmonic nanostructures^33^,^34^,^35^.

**Figure 1:**
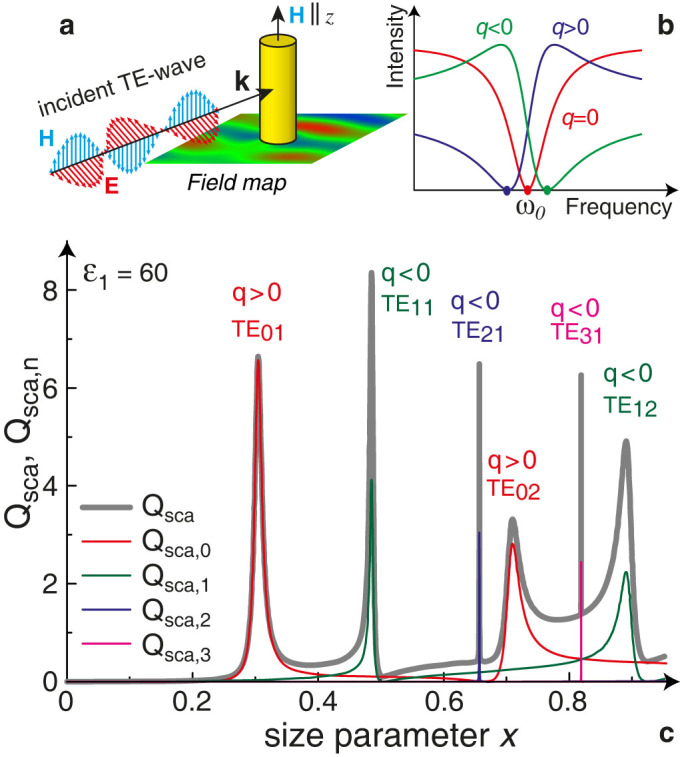
Mie scattering from a dielectric rod. (a) Schematic of the scattering geometry for TE polarization. (b) Fano lineshapes depending on the sign and value of the Fano parameter *q*. (c) Spectra of the Mie scattering efficiency *Q*_sca,n_ for dipole and multipole modes TE*_nk_*, and for the total scattering efficiency *Q*_sca_ of a single dielectric circular rod with *ε*_1_ = 60 and *ε*_2_ = 1. The sign of the corresponding values of the Fano parameter *q* is shown for each Mie resonance. The size parameter *x* = *r* *ω*/*c*.

Fano resonance is observed when the wave scattering process can reach the same final state via two different paths^23^. The first scattering path corresponds to the formation of a narrow resonant band, where the wave phase changes by ≈ *π*. The second scattering path corresponds to a broad background, where the wave phase and amplitude are nearly constant in the spectrum range of interest. The resonant band can be described as a complex Lorentz function *L*(**Ω) = (Ω + *i*)^−1^, where Ω = (*ω* − *ω*_0_)/(Γ/2), while *ω*_0_, and Γ correspond to the position and the width of the band. The continuum is defined as *B* exp(*iφ_B_*). The resulting wave takes a form *A* exp (*iφ_A_*)/(Ω + *i*) + *B* exp (*iφ_B_*) where *A*(*ω*), *B*(*ω*), *φ_A_*(*ω*) and *φ_B_*(*ω*) are real functions, frequency dependence of which can be neglected compared to the Lorentz function. If there is no parts of background avoiding the above interaction, following Fano relation takes the form
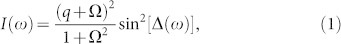
where *q* = cot Δ is the Fano asymmetry parameter, sin^2^[D(*ω*)] represents a background produced by a plane wave, Δ(*ω*) = *φ_A_*(*ω*) − *φ_B_*(*ω*) is the phase difference between the narrow resonant and continuum states, *B*(*ω*) = sin[Δ(*ω*)] At *q* = 0, instead of the conventional Mie peak, the resonant Mie dip with symmetrical Lorentzian shape *I*(*ω*)~Ω^2^/(1 + Ω^2^) is observed with the scattering intensity vanishing at the eigenfrequency *ω*_0_. Destructive interference results in complete suppression of the scattering intensity at a given frequency^18^. When the Fano parameter *q* deviates from zero, the Fano lineshape becomes asymmetric, and the zero-intensity frequency becomes shifted away from the resonance *ω*_0_. It is important to emphasize that *at any finite value of q there exists zero-intensity frequency* *ω*_zero_ = *ω*_0_ − *q*Γ/2, see Eq. (1). Note that *ω*_zero_ < *ω*_0_ at *q* > 0 and *ω*_zero_ > *ω*_0_ at *q* < 0 that is clearly seen in Fig. 1b. We employ this property of the Fano lineshape in order to realize the invisibility cloaking for a high-index dielectric rod in free space.

### Fano resonances in the Mie scattering

Our study outlined below is limited to the case of an infinitely long dielectric circular rod. However, the suggested concept and subsequent analysis are rather general, and they can be applied to other types of “bodies of revolution”. The case of an infinite rod, referred to as the Lorenz-Mie theory, corresponds to a two-dimensional problem of scattering in the plane normal to the symmetry axis *z*, and it involves cylindrical rather than spherical functions in the infinite series of the analytical Mie solution^20^,^21^,^22^.

We consider the Mie scattering by a single homogeneous infinite circular rod with the radius *r* and the purely real dielectric permittivity *ε*_1_ embedded in the transparent and homogeneous surrounding medium with the dielectric permittivity of *ε*_2_ = 1. The Mie scattering by a cylinder can be expanded into orthogonal electromagnetic dipolar and multipolar terms, with cylindrical Lorenz-Mie coefficients *a_n_* and *b_n_*^21^. For the TE-polarization considered here, the scattered fields are defined by coefficients of only one type *a_n_*, while *b_n_* are equal to zero. In addition, we introduce new coefficients *d_n_* to characterize the field inside the cylinder; more details are given in Methods. The resonances are denoted as TE*_nk_* where *n* is multipole order, and *k* is resonance number (*n* is integer and *k* is positive integer).

Figure 1c presents the total Mie scattering efficiency 
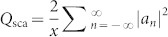
 and the spectral dependence of the Mie scattering efficiency of individual modes 
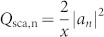
 in the low-frequency part of the spectrum at *ε*_1_ = 60 where we observe the modes TE_0*k*_, TE_1*k*_, TE_2*k*_ and TE_3*k*_. Intensity of the multipole modes TE_4*k*_, TE_5*k*_, (*n* > 3) differs from zero for higher frequencies for *x* > 1. Spectra of the Mie scattering presented in Fig. 1c demonstrate different possible lineshapes that can be described by the Fano formula (1). Namely, TE_01_ mode is quite symmetrical (|*q*| is big enough), while TE_02_ mode has typical Fano lineshape with negative *q*. Both TE_1_ modes have Fano lineshape with negative *q*. The higher order modes (TE_2_ and TE_3_) are very narrow in the long wavelength spectral range, however it is straightforward to identify *q*-parameter for these peaks.

**Figure 2:**
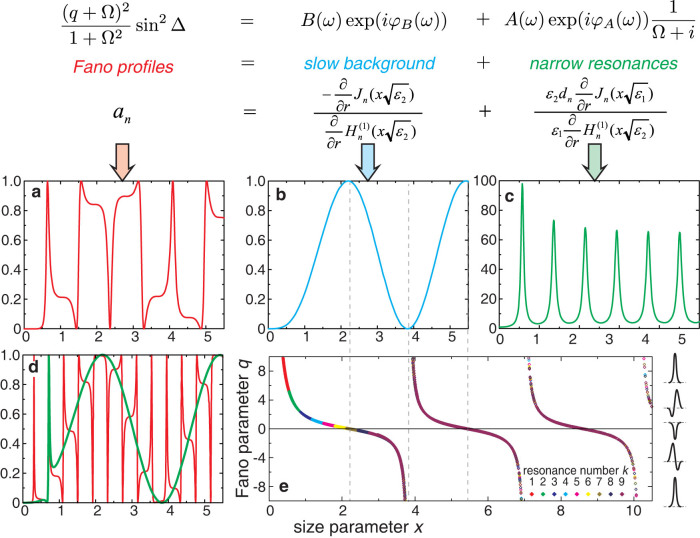
Fano resonances for the Mie resonant modes. (a) Spectra of squared modules of the Lorenz-Mie coefficient |*a*_0_|^2^ describing magnetic field outside a circular rod with *ε*_1_ = 16. (b) The background spectrum. (c) Spectra of squared modules of the Lorenz-Mie coefficient |*d*_0_|^2^ describing magnetic field inside a rod with *ε*_1_ = 16. (d) Squared Lorenz-Mie coefficient |*a*_0_|^2^ for a rod with *ε*_1_ = 60 and an example of the Fano fitting of the TE_02_, mode (green curve). (e) Calculated dependence of the Fano parameter *q* on the size parameter *x* = *rω*/*c* = 2*πr*/*λ* for the dipole modes TE_0*k*_ (1 ≤ *k* ≤ 9) of a circular rod (1 ≤ *ε*_1_ ≤ 150) embedded in air (*ε*_2_ = 1). Four characteristic Fano lineshapes for selected values of *q* are shown on the right. **Top** The equation expressed the Fano resonance condition and the Maxwell boundary condition. *J_n_*(*ζ*) and 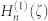 are the Bessel and Hankel functions.

We show in Fig. 2 and in Methods how the spectrum of the Mie scattering can be presented in the form of an infinite series of the Fano profiles. We consider the Maxwell boundary condition for the tangential components of *E* field at the cylinder surface for the TE polarization The interference of the background and resonant scattered fields creates a cascade of Fano profiles (see Fig. 1c or 2). In contrast to the spectra of |*d*_0_|^2^ describing magnetic field inside the rod, the scattering spectra outside the rod |*a*_0_|^2^ demonstrate asymmetric profiles with either sharp increase or drop of |*a*_0_|^2^ values at the resonance frequencies of the cylinder eigenmodes. To demonstrate that we really have the Fano resonance, we calculated the spectral dependence of the Fano parameter *q* for the dipole mode TE_0*k*_ using the profiles of 2700 resonances (for 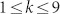 and for 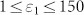 with the step of Δ*ε* = 0.5) in the broad spectral range at *ε*_2_ = 1. Figure 2d shows an example of such a fitting for TE_02_ mode at *ε*_1_ = 60 with Fano parameter *q* = 3.55 at *x* = 0.71. Finally we obtained an important result: all 2700 values of the Fano parameter *q* forms a general dependence *q*(*x*) (Fig. 2e). As follows from the fitting results, the roots of the background function define the points of infinity *q*(*x*) → ±∞ while the maxima of the background define the roots of *q*(*x*) (see common vertical dashed lines in Figs. 2b and 2e). The Fano parameter demonstrates characteristic cotangent-type dependence that is the key feature of the Fano approach demonstrating that the Fano lineshape depends only on the position of the resonance on the frequency scale with respect to the maximum (or minimum) of the background. Each Mie resonance is phase shifted with respect to the maximum of the sin - type background by phase Δ(*ω*). In particular, Mie resonances positioned close to the background maxima (at *x* ≈ 2.2, 5.4 etc) exhibit symmetric Lorentzian-type dips while Mie resonances positioned close to the background minima (at *x* = 0, ≈ 3.8 etc) exhibit symmetric Lorentzian-type peaks. On the both sides of the maxima mirror-like asymmetric resonance profiles are observed. This law is valid independently on the rod permittivity *ε*_1_.

**Figure 3:**
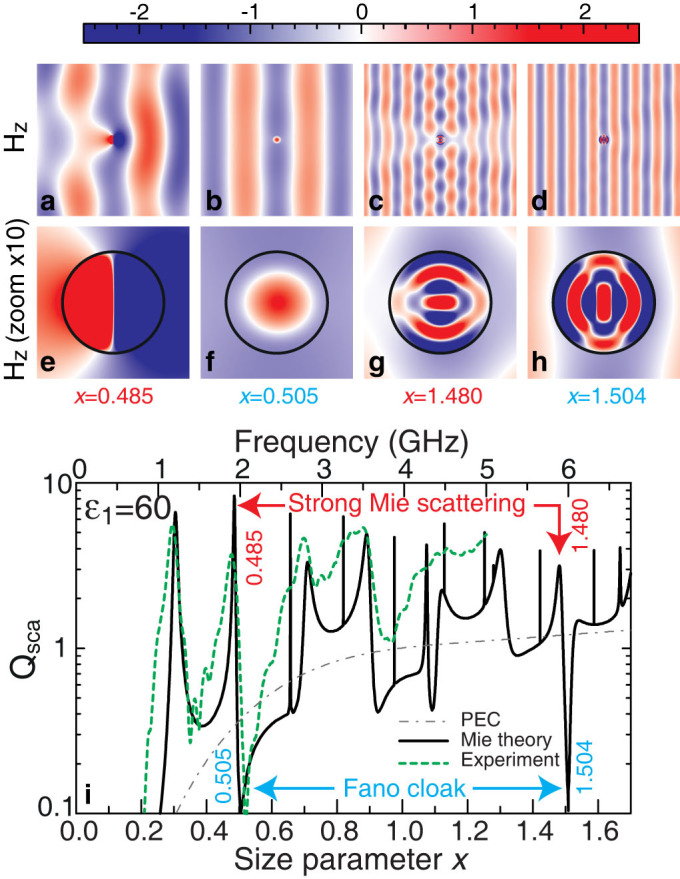
Magnetic field map and scattering efficiency spectra for TE polarization. (a–h) Results of numerical calculations for the Mie scattering by a single cylinder (*ε*_1_ = 60) embedded in air (*ε*_2_ = 1). (a–h) *H_z_* component of the TE polarized electromagnetic field (a–d) around cylinder and (e–h) inside cylinder. Shown are: strong Mie scattering (uncloaked) regimes at (a,e) *x* = 0.485 and (c,g) *x* = 1.48, as well as Fano cloaking regimes at *x*_cloak_ = 0.505 (b,f) and *x*_cloak_ = 1.504 (d,h). (i) Spectral dependence of the scattering efficiency *Q_sca_* (black curve), scattering efficiency of the perfect metallic conductor (gray dash-and-dot curve), and the experimentally measured scattering efficiency of water's cylinder in free space (green dashed curve).

Numerically calculated structure of the magnetic field in the regimes of Fano cloaking and strong Mie scattering are shown in Fig. 3a–h. We observe a practically complete suppression of scattering at frequencies corresponding to the dips in the function *Q*_sca_(*x*) (Fig. 3i. It means that the incident TE-polarized light passes the cylinder without scattering making the cylinder invisible from *any angle of observation*. To analyze the invisibility dynamics, we calculated the dip intensity as a function of dielectric constant *ε*_1_ for the lowest dip in the frequency scale. For a reference scattering intensity we choose standard scattering efficiency for cylinder made from perfect conducting metal (PEC). The calculations show that the scattering efficiencies of dielectric cylinder and PEC coincide when cylinder permittivity is about 10. At *ε*_1_ > 10 the invisibility dynamics appears for *x* around 0.505.

### Experimental verification of tunable invisibility

In the case of high-index dielectric materials and weak losses, a typical asymmetric Fano profile has a local maximum and a local minimum located close to each other, as shown in Fig. 3i. The existence of the first strong dip at *ω*_cloak_ = 0.505*c*/*r* (*ε*_1_ = 60) is caused by several reasons: the perfect zero-intensity condition at *ω*_zero_ > *ω*_0_ (*q* < 0) for nearby Fano-type TE_11_ mode, narrowness of the neighboring TE_21_ mode and long-distance location to two intense scattering bands TE_01_ and TE_02_ (Fig. 1c). This proximity can be employed for the demonstration of the switching between visability and invisibility, tuning the scattering from the uncloaked to cloaked regimes. This can be achieved by modulating the parameters of an object or by changing the wavelength of the incoming radiation. To demonstrate the concept of Fano cloaking in experiment, we employ the advantages of strong temperature dependence of the dielectric permittivity of water^36^.

At microwave frequencies, we use a glass cylinder filled with water that is characterized by dielectric constant of *ε* = 80 at 20°C and *ε* = 50 at 90°C in the frequency range from 1 GHz to 6 GHz. A rectangular horn antenna (TRIM 0.75 GHz to 18 GHz; DR) connected to a transmitting port of the vector network analyzer Agilent E8362C is used to approximate a plane-wave excitation. The water cylinder with radius 12 mm and height 42 cm is placed into the far-field region of the antenna (on the distance approximately 2.5 m) and the similar horn antenna (TRIM 0.75 GHz to 18 GHz) is employed as a receiver. The scattering efficiency is yielded from the imaginary part of the forward scattering amplitude (due to the optical theorem). The latter is proportional to *E*/*E*_0_ − 1, where *E*_0_ is measured electric field in the free space, and *E* is the electric field in the presence of the cylinder with water. Note that the dielectric permittivity of the glass tube is much smaller than that for water for microwaves and the tube has no effect on the measured scattering efficiency.

**Figure 4:**
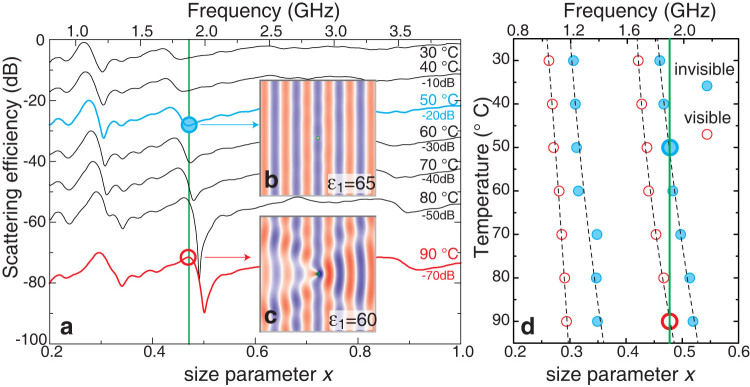
Experimental demonstration of tunable invisibility. (a) Measured temperature dependence of the scattering efficiency of a glass tube filled with water. Curves are shifted vertically by the values marked on the plot. Inserts (b,c) show the calculated magnetic fields at the frequency 1.9 GHz in the regimes of the Fano invisibility and strong Mie scattering, respectively. (d) Measured positions of dips in the scattering efficiency, corresponding to invisible cylinder, and peaks, corresponding visible cylinder and strong Mie scattering. Dashed lines are result of linear fitting to the measured maxima and minima positions.

Figure 3i shows strong suppression of scattering (of the order of 20 dB) from a glass tube filled with water and measured in microwave experiment; the data agree well with the theoretical predictions. A strong temperature dependence of the dielectric permittivity of water *ε*_1_ leads to a profound shift of the scattering maxima (the uncloaked regime) and minima (the cloaked regime) located in the spectrum near the resonant frequencies of the Mie resonance modes TE_01_ (the spectral region *ω*~ 1.25 GHz) and TE_11_ (*ω* ~ 2 GHz), as shown in Fig. 4.

## Discussion

Fano resonance is known to be extremely sensible to variation of parameters due to proximity of the maxima and minima in the frequency scale. We have changed the rod permittivity to shift TE_11_ resonance position. When sharp wing of Fano lineshape moves through a fixed frequency we observe a dramatic change of scattering intensity from a high to negligible value. Here the Fano resonance allows us to switch a glass tube with water from the visible regime of strong Mie scattering (*T* = 90°C) to the regime of strong invisibility (*T* = 50°C) *at the same frequency of 1.9 GHz*. Numerical calculations of the component *H_z_* of the electromagnetic field confirm directly the switching effect, and demonstrate both perfect invisibility in Fig. 4b,d and strongly distorted scattered fields in Fig. 4a,c.

So, we have presented the first experimental observation of tunable invisibility of a macroscopic object transformed from visible to invisible states and back, without any coating layers. We have shown that the total intensity of the Mie scattering for waves of a certain polarization vanishes under the condition of the Fano resonance at any angle of observation. Remarkable that high-index dielectric materials are available for different wavelength ranges or can be engineered at will^37^. Our study reveals a novel physics behind the seemingly well-known Mie scattering of light and it may open a novel route towards manipulation and control of electromagnetic waves in all-dielectric nanophotonics.

## Methods

### Fano formula

First, we consider a classical problem of the Fano resonance that appears as a result of interference between a narrow (resonant) band and a continuum background known as the configuration interaction in the physics of quantum phenomena. The resonant band can be described by a complex Lorentzian function *L*(Ω) = (Ω + *i*)^−1^, where Ω = (*ω* − *ω*_0_)/(Γ/2), while *ω*_0_, and Γ corresponds to the position and width of the narrow frequency band.

We can define the continuum spectrum as *B* exp(*iφ_B_*), so that the resulting combined wave takes the form
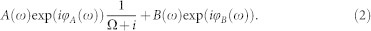
Here *A*(*ω*), *B*(*ω*), *φ_A_*(*ω*), and *φ_B_*(*ω*) are real functions which relative changes in the frequency range of interest are negligible in comparison with the Lorentzian function. The intensity of the resulting wave is given by
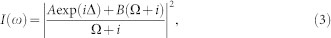
where Δ(*ω*) = *φ_A_*(*ω*) − *φ_B_*(*ω*) is the phase difference between the resonant and continuum states. The resulting extended Fano formula can be written in the form
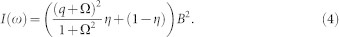


In Eq. (4), the parameter *q* is defined as the Fano asymmetry parameter that characterizes a relative transition strength for the discrete state vs. continuum set of states. The first term in Eq. (4) describes a narrow band, and the additional background spectrum in the region of the narrow band is presented by the second term. The background component that does not interfere with the narrow band is accounted for by the introduction of an interaction coefficient *η* ∈ [0..1]. Such non-interacting background is observed experimentally in the light scattering from different physical systems^28^,^29^,^30^ and photonic crystals^31^,^38^. Comparing Eq. (3) and Eq. (4), we obtain
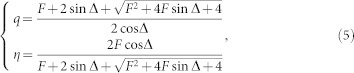
where *F* = *A*/*B* is the relative intensity of the narrow band and background component.

For *η* = 1 and *A* = 1, we obtain the Fano lineshape profile,
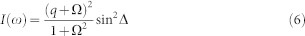
where *q* = cotΔ and *B* = sinΔ. We notice that the absolute value of the Fano parameter *q* is a measure of the relative strength of the amplitude of the resonant scattering compared to its nonresonant value.

### Mie scattering as a cascade of Fano resonances

To demonstrate that the spectrum of Mie scattering can be presented in the form of an infinite series of the Fano profiles, we consider elastic scattering of electromagnetic waves by a homogeneous infinite dielectric rod of the radius *r* and the purely real dielectric permittivity *ε*_1_. The surrounding medium have the dielectric permittivity of *ε*_2_. The far-field scattered by a cylindrical rod can be expanded into orthogonal electromagnetic dipolar and multipolar terms, with cylindrical Lorenz-Mie coefficients *a_n_* and *b_n_*^21^. For the TE-polarization the scattered fields are defined by coefficients of only one type *a_n_*, while *b_n_* are equal to zero. The Maxwell boundary conditions for the tangential components of H and E fields at the rod's surface for the TE polarization can be presented as
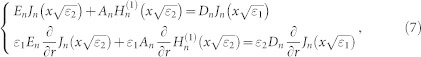
where *x* = *rω*/*c* = 2*π r*/*λ*, *E_n_*, *A_n_* and *D_n_* are cylindrical harmonic amplitudes of the incident, scattered and internal magnetic fields, respectively, expressed in terms of the Bessel *J_n_*(*ζ*) and Hankel 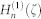 functions. The Lorenz-Mie coefficients *a_n_* = *A_n_*/*E_n_* and coefficients *d_n_* = *D_n_*/*E_n_* for the scattered and internal magnetic fields can be determined from the system (7) as:
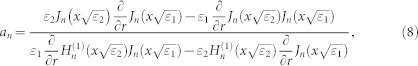

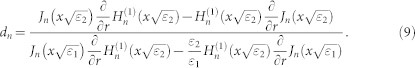


Figure 2 demonstrates that the spectrum of Mie scattering can be presented in the form of an infinite series of the Fano profiles. We write simultaneously two relations (i) the expression for the Lorenz-Mie coefficient *a_n_* which defines the scattered field in accord to (7), (ii) analytical expression describing the Fano resonance at the interference of a narrow resonance (symmetric Lorentzian) with a slow varying background (plane wave).

We identify two terms of different line-width with the first one 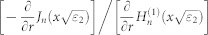 as slowly changed background and second one, 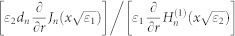 as symmetric Lorentzian, both oscillating with the resonance frequency *ω*. Therefore we have a Fano resonance between background scattering from the rod and resonant Mie mode within the same cylindrical harmonic (see Fig. 2). The interference of the incident and scattered fields creates a complicated near-field pattern, and it may give rise either to strong enhancement (constructive interference) or strong suppression (destructive interference) of the electromagnetic field around the rod.

The similar analysis can be performed in the case of the TM polarized waves and for any “body of revolution” including a sphere.

## Author Contributions

M.R. developed a theoretical model and conducted simulations and data analysis. D.F. performed experimental measurement. M.L, P.B. and Y.K. provided a guidance on the theory, numerical analysis and experiment. All authors discussed the results and contributed to the writing of the manuscript.

## References

[b1] Zheludev N. I. & Kivshar Y. S. From metamaterials to metadevices. Nature Mater. 11, 917–924 (2012). 23089997 10.1038/nmat3431

[b2] Leonhardt U. Optical conformal mapping. Science 312, 1777–1780 (2006). 16728596 10.1126/science.1126493

[b3] Pendry J. B., Schurig D. & Smith D. R. Controlling electromagnetic fields. Science 312, 1780–1782 (2006). 16728597 10.1126/science.1125907

[b4] Schurig D. *et al.* Metamaterial electromagnetic cloak at microwave frequencies. Science 314, 977–980 (2006). 17053110 10.1126/science.1133628

[b5] Cai W., Chettiar U. K., Kildishev A. V. & Shalaev V. M. Optical cloaking with metamaterials. Nature Photon. 1, 224–227 (2007).

[b6] Ruan Z. & Fan S. Temporal coupled-mode theory for Fano resonance in light scattering by a single obstacle. J. Phys. Chem. C 114, 7324–7329 (2009).

[b7] Edwards B., Alù A., Silveirinha M. G. & Engheta N. Experimental verification of plasmonic cloaking at microwave frequencies with metamaterials. Phys. Rev. Lett. 103, 153901 (2009). 19905638 10.1103/PhysRevLett.103.153901

[b8] Chen P.-Y., Soric J. & Alù A. Invisibility and cloaking based on scattering cancellation. Adv. Mater. 24, OP281–OP304 (2012). 23080411 10.1002/adma.201202624

[b9] Zharova N. A., Shadrivov I. V., Zharov A. A. & Kivshar Y. S. Nonlinear control of invisibility cloaking. Opt. Express 20, 14954–14959 (2012). 22772190 10.1364/OE.20.014954

[b10] Monticone F., Argyropoulos C. & Alù A. Multilayered plasmonic covers for comblike scattering response and optical tagging. Phys. Rev. Lett. 110, 113901 (2013). 25166536 10.1103/PhysRevLett.110.113901

[b11] Chen P.-Y. & Alù A. Atomically thin surface cloak using graphene monolayers. ACS Nano 5, 5855–5863 (2011). 21662981 10.1021/nn201622e

[b12] Kort-Kamp W. J. M., Rosa F. S. S., Pinheiro F. A. & Farina C. Tuning plasmonic cloaks with an external magnetic field. Phys. Rev. Lett. 111, 215504 (2013). 24313504 10.1103/PhysRevLett.111.215504

[b13] Zang X. F. *et al.* Rotatable illusion media for manipulating terahertz electromagnetic waves. Opt. Express 21, 25565–25572 (2013). 24150396 10.1364/OE.21.025565

[b14] Zang X. F. *et al.* Illusion induced overlapped optics. Opt. Express 22, 582–592 (2014). 24515019 10.1364/OE.22.000582

[b15] Leonhardt U. & Philbin T. G. General relativity in electrical engineering. New J. Phys. 8, 247 (2006).

[b16] Alù A. & Engheta N. Achieving transparency with plasmonic and metamaterial coatings. Phys. Rev. E 72, 016623 (2005). 10.1103/PhysRevE.72.016623 16090123

[b17] Alù A. & Engheta N. Multifrequency optical invisibility cloak with layered plasmonic shells. Phys. Rev. Lett. 100, 113901 (2008). 18517786 10.1103/PhysRevLett.100.113901

[b18] Fano U. Effects of configuration interaction on intensities and phase shifts. Phys. Rev. 124, 1866–1878 (1961).

[b19] Rybin M. V. *et al.* Mie scattering as a cascade of Fano resonances. Opt. Express 21, 30107–30113 (2013). 24514559 10.1364/OE.21.030107

[b20] Hulst H. C. & van de Hulst H. C. Light scattering: by small particles (Courier Dover Publications, New York, 1957).

[b21] Bohren C. F. & Huffman D. R. Absorption and scattering of light by small particles (Wiley-VCH, New York, 1998).

[b22] Stratton J. A. Electromagnetic theory, vol. 33 (Wiley, New York, 2007).

[b23] Miroshnichenko A. E., Flach S. & Kivshar Y. S. Fano resonances in nanoscale structures. Rev. Mod. Phys. 82, 2257–2298 (2010).

[b24] Madhavan V., Chen W., Jamneala T., Crommie M. F. & Wingreen N. S. Tunneling into a single magnetic atom: Spectroscopic evidence of the Kondo resonance. Science 280, 567 (1998). 9554843 10.1126/science.280.5363.567

[b25] Kabachnik N. M. & Sazhina I. P. Angular distribution and polarization of photoelectrons in the region of resonances. J. Phys. B 9, 1681–1697 (1976).

[b26] Hopfield J. J., Dean P. J. & Thomas D. G. Interference between intermediate states in the optical properties of nitrogen-doped gallium phosphide. Phys. Rev. 158, 748–755 (1967).

[b27] Cerdeira F., Fjeldly T. A. & Cardona M. Effect of free carriers on zone-center vibrational modes in heavily doped p-type Si. II. optical modes. Phys. Rev. B 8, 4734–4745 (1973).

[b28] Friedl B., Thomsen C. & Cardona M. Determination of the superconducting gap in RBa_2_Cu_3_O_7−*δ*_. Phys. Rev. Lett. 65, 915–918 (1990). 10043055 10.1103/PhysRevLett.65.915

[b29] Limonov M. F., Tajima S. & Yamanaka A. Phononic and electronic Raman spectroscopy of the pseudogap state in underdoped YBa_2_Cu_3_O_7−*x*_. Phys. Rev. B 62, 11859–11863 (2000).

[b30] Limonov M., Lee S., Tajima S. & Yamanaka A. Superconductivity-induced resonant raman scattering in multilayer high-T*_c_* superconductors. Phys. Rev. B 66, 054509 (2002).

[b31] Rybin M. V. *et al.* Fano resonance between Mie and Bragg scattering in photonic crystals. Phys. Rev. Lett. 103, 023901 (2009). 19659204 10.1103/PhysRevLett.103.023901

[b32] Poddubny A. N., Rybin M. V., Limonov M. F. & Kivshar Y. S. Fano interference governs wave transport in disordered systems. Nature Commun. 3, 914 (2012). 22735442 10.1038/ncomms1924 PMC3621451

[b33] Tribelsky M. I., Flach S., Miroshnichenko A. E., Gorbach A. V. & Kivshar Y. S. Light scattering by a finite obstacle and Fano resonances. Phys. Rev. Lett. 100, 043903 (2008). 18352275 10.1103/PhysRevLett.100.043903

[b34] Luk'yanchuk B. *et al.* The Fano resonance in plasmonic nanostructures and metamaterials. Nature Mater. 9, 707–715 (2010). 20733610 10.1038/nmat2810

[b35] Francescato Y., Giannini V. & Maier S. A. Plasmonic systems unveiled by Fano resonances. ACS Nano 6, 1830–1838 (2012). 22280066 10.1021/nn2050533

[b36] Zelsmann H. R. Temperature dependence of the optical constants for liquid H_2_O and D_2_O in the far IR region. J. Mol. Struct. 350, 95–114 (1995).

[b37] Hu W. *et al.* Electron-pinned defect-dipoles for high-performance colossal permittivity materials. Nature Mater. 12, 821–826 (2013). 23812129 10.1038/nmat3691

[b38] Rybin M. V. *et al.* Bragg scattering induces Fano resonance in photonic crystals. Photon. Nanostr. 8, 86–93 (2010).

